# Adaptation of the binding domain of *Lactobacillus acidophilus* S-layer protein as a molecular tag for affinity chromatography development

**DOI:** 10.3389/fmicb.2023.1210898

**Published:** 2023-06-13

**Authors:** Emanuel J. Muruaga, Paula J. Uriza, Gonzalo A. K. Eckert, María V. Pepe, Cecilia M. Duarte, Mara S. Roset, Gabriel Briones

**Affiliations:** ^1^Instituto de Investigaciones Biotecnológicas, Universidad Nacional de San Martín (UNSAM)- Consejo Nacional de Investigaciones Científicas y Técnicas (CONICET), Buenos Aires, Argentina; ^2^Escuela de Bio y Nanotecnologías (EByN), Universidad Nacional de San Martín, Buenos Aires, Argentina

**Keywords:** affinity chromatography, S-layer proteins, *Bacillus subtilis* natto, SLAP_TAG_, applied microbiology and biotechnology

## Abstract

**Introduction:**

The S-layer proteins are a class of self-assembling proteins that form bi-dimensional lattices named S-Layer on the cell surface of bacteria and archaea. The protein SlpA, which is the major constituent of the *Lactobacillus acidophilus* S-layer, contains in its C-terminus region (SlpA^284 − 444^), a protein domain (named here as SLAP_TAG_) responsible for the association of SlpA to the bacterial surface. SLAP_TAG_ was adapted for the development of a novel affinity chromatography method: the SLAP_TAG_-based affinity chromatography (SAC).

**Methods:**

Proteins with different molecular weights or biochemical functions were fused in-frame to the SLAP_TAG_ and efficiently purified by a *Bacillus subtilis*-derived affinity matrix (named Bio-Matrix or BM). Different binding and elution conditions were evaluated to establish an optimized protocol.

**Results:**

The binding equilibrium between SLAP_TAG_ and BM was reached after a few minutes of incubation at 4°C, with an apparent dissociation constant (K_D_) of 4.3μM. A reporter protein (H6-GFP-SLAP_TAG_) was used to compare SAC protein purification efficiency against commercial immobilized metal affinity chromatography. No differences in protein purification performance were observed between the two methods. The stability and reusability of the BM were evaluated, and it was found that the matrix remained stable for more than a year. BM could be reused up to five times without a significant loss in performance. Additionally, the recovery of bound SLAP-tagged proteins was explored using proteolysis with a SLAP-tagged version of the HRV-3c protease (SLAP_ASE_). This released the untagged GFP while the cut SLAP_TAG_ and the SLAP_ASE_ were retained in the BM. As an alternative, iron nanoparticles were linked to the BM, resulting in BM_mag_. The BM_mag_ was successfully adapted for a magnetic SAC, a technique with potential applications in high-throughput protein production and purification.

**Discussion:**

The SAC protocol can be adapted as a universal tool for the purification of recombinant proteins. Furthermore, the SAC protocol utilizes simple and low-cost reagents, making it suitable for in-house protein purification systems in laboratories worldwide. This enables the production of pure recombinant proteins for research, diagnosis, and the food industry.

## Introduction

Proteins are biopolymers formed by a particular amino acid sequence that determines a given atomic spatial distribution, also known as protein conformation. Far from being a static structure, proteins behave as “nano-machines” performing precise molecular activities due to internal movements of the protein parts or protein domains (Wood, 2004). In addition, proteins can interact intramolecularly or intermolecularly with different macromolecules such as proteins, DNA, RNA, polysaccharides, or small compounds (Buxbaum, 2015).

Proteins perform vital functions in life. For instance, some proteins break down food into nutrients during the process of digestion in the human body, while other proteins transport critical compounds for supporting life (like hemoglobin that transports oxygen to the cells), shape the cellular structure (like actin, tubulin, or keratin), function as hormones (like insulin), or defend the organism against pathogens (like the antibodies) (Buxbaum, 2015).

In addition to the multiplicity of existent polypeptides in nature, molecular biology and biotechnology have generated a myriad of chimeric proteins by combining different protein domains (Baldo, 2015). These artificial proteins can be used as therapeutic tools to treat cancer, autoimmunity, or different medical conditions (Wen et al., 2009).

However, for various applications such as biochemical, industrial, or medical purposes, proteins need to be purified, stabilized, and concentrated to a high degree to be useful. This condition is reached by a series of steps oriented to isolate and purify a desired protein from the other proteins in the mixture, free of contaminants, and preserving its biological activity. The purification process exploits differences in size, charge, hydrophobicity, ligand-binding affinity, or specific sequences (Schales, 1942; Freitag and Horváth, 1996; Wood, 2014). Several fractionations of chromatographic steps can be combined to efficiently enrich or purify a particular protein.

Affinity chromatography is a special type of liquid chromatography that exploits the existence of natural (Kuntz et al., 1999) or artificial (Mouratou et al., 2015) affinities between two moieties: the molecular target (or tag) and its ligand (the affinity ligand) which is usually immobilized onto a chromatographic stationary phase to generate a chromatography matrix. Thus, proteins of interest can be fused in-frame to different molecular tags (such as His-6X, GST, and MBP) expressed and purified ideally in a single step from a complex mixture of proteins. Additionally, there is a type of affinity chromatography that allows the purification of antibodies without the need for a molecular tag. This method employs proteins such as protein A, protein G, or different synthetic proteins that exhibit a high affinity for the Fc region of IgG antibodies (Mouratou et al., 2015).

Since it was developed by Starkestein in 1910 (Hais, 1986), affinity chromatography was gaining popularity and centrality for many industrial processes such as the purification of proteins for diagnosis, research, and therapeutic purposes (Rodriguez et al., 2020). Regulatory requirements for the purity and quality of the proteins vary greatly depending on the area of application. For instance, bio-products can be used with little purification for industrial use. In addition, the recombinant protein produced for research, diagnosis, or non-clinical purposes has a less stringent regulatory approval process than proteins designed as biopharmaceuticals.

The most common strategy for affinity chromatography is the ON/OFF format. In the “ON” phase, a biological sample containing the protein of interest (that can be fused in-frame to a molecular tag) is formulated in a specific application buffer that will favor the binding process to a ligand. Then, the sample is placed in contact with a chromatography material (or chromatography matrix) associated with the affinity ligand that will consequently recognize and retain the tagged protein. Finally, the chromatography matrix is washed several times to remove all the unbound protein. In the “OFF” phase (or elution phase), an elution buffer is passed or incubated to release the tagged protein by changing the pH or the ionic strength modifying the affinity of the interaction (non-specific elution) or by the addition of the free ligand that will out-compete the retained tagged protein (bio-specific elution) (Rodriguez et al., 2020). The simplicity and specificity of affinity chromatography have made this technique central for the purification of biomolecules and biopharmaceuticals.

Here, we explored the adaptation of a protein domain present in the carboxy terminus of the *Lactobacillus acidophilus* protein SlpA as a molecular tag. *In silico* analysis of this region (SlpA^284 − 444^) allowed us to identify a tandem of two copies of the 60-aminoacid protein domain named SLP-A (pfam03217) which is necessary for the association (Supplementary Figure 1). Here, this dual SLP-A domain was named SLAP_TAG_, and the adaptation as a molecular tag was evaluated. Recently, we have characterized the binding properties of the SLAP_TAG_ and characterized its association with the cell wall of live *Lactobacillus* for vaccine purposes (Uriza et al., 2020). Thus, the SLAP_TAG_ was fused in-frame to a chimeric antigen derived from the Shiga toxin-producing *Escherichia coli* (STEC) formed by the peptides EspA^36 − 192^, Intimin^653 − 953^, and Tir^240 − 378^ (or EIT). The resulting chimeric antigen (EIT-SLAP_TAG_) recombinantly expressed and purified was able to associate with the bacterial cell wall of *L. acidophilus*, a process that we named decoration. Thus, EIT-decorated *L. acidophilus* after oral administration was able to deliver the EIT antigen to the intestinal mucosa eliciting a protective immune response that controls an experimental STEC infection in mice (Uriza et al., 2020). Remarkably, the decoration process does not modify genetically the *Lactobacillus* genome preserving its GRAS (Generally Recognized as Safe) status, a trait that is important for vaccine purposes.

Here, we explore the adaptation of the SLAP_TAG_ for the development of novel affinity batch chromatography, the SLAP affinity chromatography (SAC), and a comprehensive protocol is presented.

## Materials and methods

### Strains and plasmids

All the bacterial strains and plasmids used here are summarized in Table 1. Bacteria *Escherichia coli* and *Bacillus subtilis* strains were grown in Luria Bertani (LB) medium (Sigma, St. Louis, MO, the United States) at 37°C and 180 rpm. Bacterial plasmid vectors were transformed into *E. coli* DH5α or *E. coli* BL21 (DE3) for protein expression. *Pichia pastoris* was grown in Yeast Extract–Peptone–Dextrose (YPD) medium at 28°C and 180 rpm. HEK293F cells were maintained at 37°C in a 5% CO2 atmosphere in Dulbecco modified Eagle medium (DMEM) supplemented with 5% fetal bovine serum and streptomycin (50 μg/ml)–penicillin (50 U/ml). The SARS-CoV-2 Spike ectodomain Hexa-pro construction (Table 1) was a gift from Jason McLellan (Addgene # 154754; http://n2t.net/addgene:154754; RRID: addgene_154754) (Hsieh et al., 2020).

**Table 1:** Bacterial strains and plasmids used in this study.

**Strain**		
*E. coli* DH5α	F–ϕ80*lac*ZΔM15 Δ(*lac*ZYA-*arg*F) U169 *rec*A1 *end*A1 *hsd*R17(rK–, mK+) *pho*A *sup*E44λ- *thi*-1 *gyr*A96 *rel*A1	Invitrogen
*E. coli* BL21 Codon plus	[*omp*T *hsd*S(rB– mB–) *dcm* + Tcr *gal* λ (DE3) *end*A Hte Cmr]	Stratagen
*B. subtilis*	Wild type strain var. *natto*	ATCC 15245
*Pichia pastoris*	*P. pastoris* strain GS115	Bio-Rad, USA
**Plasmid**		
pET28-eGFP-SlpA	A vector containing the GFP gene from *Aequorea victoria* fused to SLAP_TAG_	(Fina Martin et al., 2019)
pET22-eGFP-SlpA	A vector containing the GFP gene from *Aequorea victoria* fused to LEVLFQGP sequence and SLAP_TAG_	This study
pGEX-SlpA	A vector containing GST form *Schistosoma japonicum* fused to SLAP_TAG_	(Uriza et al., 2020)
pLC3-EITH7-SlpA	A vector containing EspA-Intimin-Tir fused genes from STEC *E. coli* fused to SLAP_TAG_	(Uriza et al., 2020)
pLC3-Omp19-SlpA	A vector containing the Omp19 gene from *Brucella abortus* fused to SLAP_TAG_	(Uriza et al., 2020)
pLC3-FliC-SlpA	A vector containing the FliC gene from *Salmonella enterica* fused to SLAP_TAG_	(Uriza et al., 2020)
pLC3-Gal8-SlpA	A vector containing the Gal8 gene from *Mus musculus* fused to SLAP_TAG_	(Uriza et al., 2020)
pET28-Lys-SlpA	A vector containing the Lysozyme gene from *Gallus* fused to SLAP_TAG_	This study
pMAL-c5X-HRV3c-SlpA	A vector containing human rhinovirus (HRV) type 14 3C protease gene fused to SLAP_TAG_ (SLAPase)	This study
pGEX-2T-EspA-SlpA	A vector containing the EspA gene from STEC *E. coli* fused to SLAP_TAG_	This study
pPICZalphaB-HRV3c-SlpA	A vector containing human rhinovirus (HRV) type 14 3C protease gene fused to SLAP_TAG_ (SLAPase)	This study
Addgene #154754	A vector containing the sequence of SARS-CoV-2 Spike ectodomain Hexa-pro	(Hsieh et al., 2020)
pSpike-SLAP_TAG_	Plasmid Addgene #154754 fused with the SLAP_TAG_	This study
pLysS	Vector for expression of T7 lysozyme.	Novagene

### Cloning

Lysozyme gene was amplified from the pLysS plasmid (Millipore Sigma, Novagen) with the oligonucleotide primers Fw-lys-*Nde*I (CCCATATGG CTCGTGTACAGTTTAAACAACGTG) and Rv-lys-SalI (CGGTCGACTCCACGGTCAGAAGTGACCAGTTCG). The PCR product was digested with the restriction enzymes *Nde*I and *Sal*I and then cloned into the pLC3-EITH7-SlpA vector in the same restriction sites, and the recombinant plasmid was transformed into *E. coli* DH5α. In addition, the lysozyme gene was subcloned into pET28-e GFP-SlpA at the *Nde*I and *Not*I restriction sites and transformed into *E. coli* BL21 by electroporation.

*EspA* gene was amplified with the following primers: *pRvEspAXbaI* (GCTCTAGATTTACCAAGGGATATTGCTG) *and pFwEITSalI* (ACGCGTCGACGATATGAATGAGGCATCTAAA). After digestion with *Xba*I and *Sal*I, the gene was cloned into *pGEX-SlpA* (Table 1), which was previously digested with the same enzymes. The plasmid was introduced by electroporation into *E. coli* BL21.

The gene encoding the human rhinovirus (HRV) type 14 3C protease fused to SLAP_TAG_ was synthesized and cloned in pMAL-c5X and pPICZ alpha B for protein expression in *E. coli* and *P. pastoris*, respectively (Gene Universal, Delaware—USA).

For *P. pastoris* GS115 transformation, 5 μg of pPICZ alpha B—*hrv-3c* plasmid was linearized with *Sac*I restriction enzyme and transformed into cells through electroporation using 2 mm gap cuvettes (1,500 V, 125 X, 50 lF). Transformed cells were selected by plating on a YPD medium containing Zeocin 100 ug/ml for resistance selection. Isolated colonies were further grown in test tubes containing YPD broth, and after 24 h, protein expression was induced by adding 1% (v/v) pure methanol every 24 h. The supernatants were sampled after 72 h of induction, and the best producer clones were chosen for further experiments.

The pET28-eGFP-SLAP was digested with *BamH*I and *Xho*I restriction enzymes, and the SLAP_TAG_-containing band was subcloned into SARS-CoV-2 S HexaPro plasmid (Addgene#154754) with the same restriction sites.

### Protein expression

*E. coli* BL21 were grown at 37°C and 180 rpm in liquid LB supplemented with antibiotics until they reached OD_600_ = 0.6. Then, 0.1 mM IPTG was added, and bacteria were grown for another 20 h at 18°C and 180 rpm. Finally, bacteria were harvested and lysed by sonication. The bacterial lysates were clarified by centrifugation.

*For Pichia, pastoris* protein expression cells were induced with methanol. In brief, *P. pastoris* cultures were grown in 50 ml of YPD for 48 h until the dextrose was consumed. Then, methanol pulses (200 ul) were supplied every 24 h for 5 days. Finally, *P. pastoris* were harvested, and the supernatants were collected.

HEK293 cells were transfected using polyethyleneimine (PEI) for protein expression. In brief, 30,000 cells per well were seeded in 24 well plates and incubated at 37°C, 5% CO_2_ for 24 h. For transfection, 4 μl of PEI was diluted in 40 μl of DMEM. 500 ng of DNA was added, and the transfection mix was incubated for 20 min at room temperature. Then, the transfection mix was transferred to the cells and incubated for 48 h. Finally, the supernatant was collected to check protein expression.

### 6xHis-tag purification method

Purification of 6xHis-tagged proteins was carried out according to the manufacturer's protocol. In brief, benchtop columns were equilibrated with the binding buffer (200 mM NaCl, 50 mM Tris-HCl pH 7.5). Columns were loaded with the bacterial lysate. After being washed, columns were eluted in steps with 10-, 50-, 100-, 300-, and 500-mM imidazole in the equilibration buffer. When compared with the SAC, the IMAC batch format was adopted. Thus, Ni-NTA superflow resin (Qiagen) was equilibrated with buffer (200 mM NaCl, 50 mM Tris-HCl pH 7.5) in 1.5 ml tubes. Cleared bacterial lysates were loaded and mixed in an orbital shaker for 1 h at 4°C. The samples were centrifugated, and, after being washed, the resin was eluted with 500-mM imidazole in an equilibration buffer.

### Bio-Matrix preparation

*B. subtilis* natto was grown in 200 mL of Luria Bertani broth at 37°C and 180 rpm for 48 h. Then, the culture was centrifugated, and the bacteria were washed twice with PBS. The culture was resuspended in PBS with 2% glutaraldehyde and incubated overnight with soft agitation. Next, fixed bacteria were washed twice with PBS and stored in 20 % ethanol. 1 mL of BM correspond to DO600 = 30 of B. subtilis.

BM was weighted in a drying scale (KERN MLS-D), and a calibration curve for dry weight vs. optical density was performed.

### SLAP_TAG_ purification method

To establish an optimized SAC protocol, BM was equilibrated in an initial binding buffer (50 mM Tris-HCl, pH 7.5). Then, the samples containing SLAP-tagged proteins were incubated with the BM at different times and temperatures. After incubation, the samples were washed three times by centrifugation (8,000 rpm) using the binding buffer. Finally, as shown in the results section, elution was evaluated by incubating at different times and temperatures with different elution buffers. Based on our experimental optimization studies, we have determined that the optimal binding conditions are 200 mM NaCl and 50 mM Tris-HCl at pH 7.5 and a temperature of 4°C for 5 min. For the elution step, we found that using a carbonate-bicarbonate buffer (0.1M) with a pH of 10 and a concentration of 200 mM NaCl for 5 min was optimal.

Once the protocol was set, proteins expressed in *Escherichia coli*, HEK293, and *Pichia pastoris* were purified from bacterial lysate or cellular supernatant using the final protocol described here.

### Bio-Matrix time stability and reusability

To analyze stability in time, aliquots of BM were frozen at −20°C. At different times, the samples were unfrozen and used for purifying GFP-SLAP following the protocol developed in this study. To analyze reusage, an aliquot of BM was used for purifying GFP-SLAP following the protocol developed in this study. After elution, the BM was washed with two volumes of the elution Buffer (0.1M carbonate-bicarbonate buffer) with a pH of 10 and a concentration of 200 mM NaCl and then two volumes of binding buffer (200 mM NaCl, 50 mM Tris-HCl pH 7.5). The cleaning process was repeated each time after elution.

### Protein analysis

Protein samples were dissolved in a cracking buffer and incubated for 5 min at 100°C. Protein electrophoresis was performed at 120 V on 12% SDS-PAGE gel. Gels were stained in a Coomassie Blue solution (20% methanol and 10% acetic acid).

For Western blot analysis, proteins were transferred to a nitrocellulose membrane for 55 min at 15 V using a semi-dry electroblotting transfer unit (Bio-Rad, Hercules, CA, the United States). Membranes were incubated for 1 h with blocking buffer (1% dry skim milk and 0.1% Tween in PBS). Then, membranes were incubated for 1 h with primary antibody diluted in blocking buffer (1/500). After washing with PBS-0.1% Tween, membranes were incubated for 1 h with IRDye fluorophore-labeled secondary antibodies (LI-COR, Lincoln, NE, the United States) diluted in a blocking buffer (1/20,000). Finally, membranes were scanned using the Odyssey Imaging System (LI-COR).

### Fluorescence measurements

GFP fluorescence measurements were performed at 485/535 nm excitation–emission wavelength, respectively, using FilterMax F5 Microplate Reader in Black 96 Well Plates (Thermo).

### Protein modeling

(H_6_)-GFP-SLAP_TAG_ structure was predicted by AlphaFold2 (Jumper et al., 2021). ColabFold web interface was employed using standard settings (five models and no templates).

### Adsorption isotherm

Adsorption isotherms for (H_6_)-GFP-SLAP_TAG_ on BM were performed using batch experiments. BM was equilibrated with binding buffer (50 mM Tris-HCl, 200 mM NaCl, and pH 7.6). Purified (H_6_)-GFP-SLAP_TAG_ protein at 3 mg/ml in binding buffer was used as a stock solution. Different concentrations of protein were incubated with 10 μl of BM. After reaching equilibrium, the samples were centrifugated and GFP fluorescence from the supernatant was measured. Unbound protein in equilibrium with the BM was calculated using GFP fluorescence. Bound protein was estimated by the difference between input and unbound protein. The adsorption isotherm data were then fitted to the Langmuir isotherm equation to calculate the parameters Qmax and K_D_.

### Confocal fluorescence microscopy

The samples were incubated for 30 min in plates treated with poly-L-Lysine (Sigma, St. Louis, MO, the United State). After treatment with PFA (4% in PBS), the samples were washed twice with PBS. Finally, the samples were observed with a confocal laser-scanning microscope Olympus FV1000 using a PlanApo N (60 × 1.42 NA) oil objective.

### Cleavage of SLAP_TAG_ with SLAP_ASE_ protease

Cleavage buffer recommended for commercial HRV3c protease was prepared: 50 mM Tris-HCl, pH 7.0, 150 mM NaCl, 1 mM EDTA, and 1 mM dithiothreitol. GFP-LEVLFQGP-SLAP_TAG_ protein was bound to the BM. After binding, BM was washed with the same buffer thrice at 4°C. SLAP_ASE_ was added and incubated at 4°C for 60 min. Percolate containing GFP protein without SLAP_TAG_ was recovered. As a control, the protease and the cut SLAP_TAG_ were eluted with Bio-Matrix elution buffer after cleavage using 2M LiCl.

### Synthesis of iron nanoparticles

Iron nanoparticles were synthesized by reverse co-precipitation as described by Nadi et al. (2019). In summary, the precursor was prepared by dissolving 0.89 g of FeCl2.4H2O in 90 mL of water. The sample was incubated in stirring for 15 min and sonicated for 10 min to ensure complete dissolution of the salt. The solution was then poured over a solution of ammonium hydroxide diluted 1:2 with water and stirred for an hour. Finally, the sample was washed repeatedly with deionized water.

### Magnetic Bio-Matrix generation

In brief, 5 ml of Bio-Matrix were resuspended in PBS and iron nanoparticles were added at a final concentration of 40 g/L. The sample was mixed with gentle stirring for 30 min. Then, it was washed repeatedly with PBS 1X and preserved in 20% ethanol.

### Antibody generation

BALB/c mice were immunized intraperitoneally with 10 μg of the different purified recombinant proteins (GST-SLAP_TAG_ or GFP) using aluminum hydroxide as an adjuvant. Boosters with 5 μg of protein were further performed at 2 and 4 weeks. One week after the last immunization, the mice were bled, and the serum was stored at −20°C for later use.

### Statistical analysis

Statistical analyses were performed using GraphPad Prism 9 software. Statistical significance was analyzed by one-way ANOVA with Bonferroni.

## Results

### SLAP_TAG_ binds rapidly and efficiently to the Bio-Matrix (BM)

**Figure 1:**
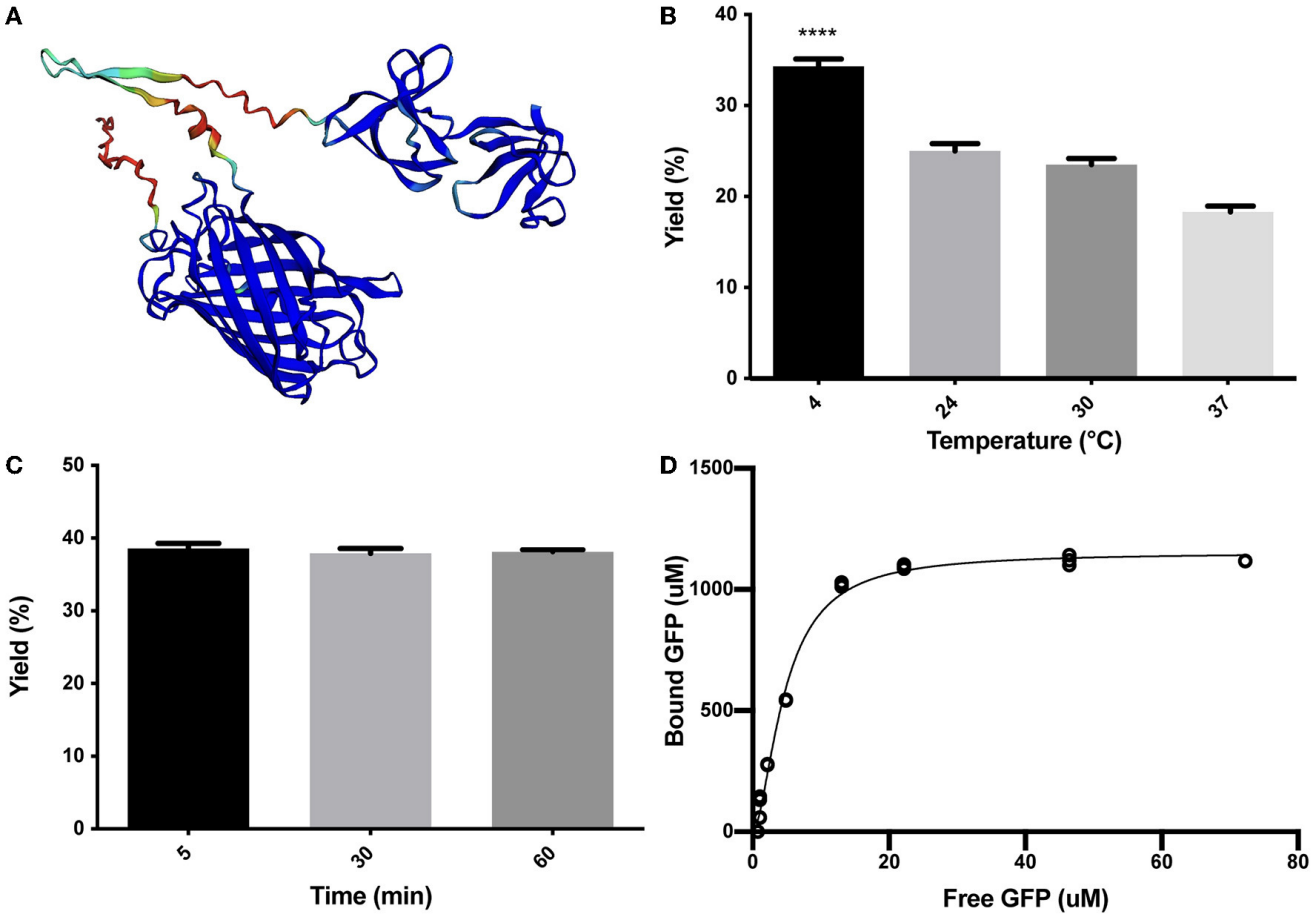
Characterization of (H_6_)-GFP-SLAP_TAG_ binding to Bio-Matrix. **(A)** The AlphaFold2 model of (H_6_)-GFP-SLAP_TAG_ fusion protein. AlphaFold2 predicted structure was automatically colored by the pLDDT confidence measure. High accuracy is colored in blue, while low accuracy is in red. Although SLAP_TAG_ has not been crystallized, structure prediction showed a good performance. **(B)** Figure compares (H_6_)-GFP-SLAP_TAG_ purification yield of SLAP_TAG_-based affinity chromatography for different temperatures of the binding process. **(C)** Figure compares (H_6_)-GFP-SLAP_TAG_ purification yield of SLAP_TAG_-based affinity chromatography for different times of binding incubation. **(D)** Adsorption isotherm of (H_6_)-GFP-SLAP_TAG_ onto Bio-Matrix. Asterisk (****) denotes significant differences using the ANOVA method, Bonferroni test (*p* < 0.0001).

As described in the Methods section, the SLAP_TAG_ (Supplementary Figure 1) was fused in-frame to the carboxy terminus of the green fluorescent protein (GFP) to generate the chimeric protein (H_6_)-GFP-SLAP_TAG_ adapted here as a SLAP_TAG_ reporter protein. As shown in Figure 1A, the structure of the chimeric protein (H_6_)-GFP-SLAP_TAG_ was predicted based on the AlphaFold2 method (Jumper et al., 2021). The beta-barrel corresponding to GFP and the two globular domains corresponding to SLAP_TAG_ were predicted with high performance, and as expected, linkers and 6xHis-tag, which are flexible regions, showed low prediction values.

As a working chromatographic matrix, a culture of *Bacillus subtilis* natto was processed as described in the Materials and Methods section to generate a bacterial-derived affinity chromatography matrix, named here Bio-Matrix (BM). Interestingly, although *B. subtilis* has no S-layer, we have demonstrated previously that this bacterium can be externally covered with SLAP-tagged proteins to generate a recombinant S-layer on its bacterial cell wall, a process that we called decoration (Uriza et al., 2020). Decoration of *B. subtilis* with SLAP-tagged proteins is possible because teichoic acid and lipoteichoic acid (which are the molecules responsible for the SLAP_TAG_ association) have the same chemical composition as *Lactobacillus acidophilus*. Interestingly, in *B. subtilis* and *L. acidophilus*, teichoic acid and lipoteichoic acid are distributed homogeneously on their cell wall.

To characterize the binding properties of the SLAP_TAG_, the reporter protein (H_6_)-GFP-SLAP_TAG_ was recombinantly expressed in *Escherichia coli*, purified by affinity chromatography mediated by its Hisx6 tag (H_6_), and further incubated with BM under a variety of conditions. Initially, as shown in Figure 1B, the optimal binding temperature of (H_6_)-GFP-SLAP_TAG_ to the BM was evaluated, finding that the best binding efficiency was reached when the incubation was performed at 4°C. To get insights into the SLAP_TAG_ association dynamics, (H_6_)-GFP-SLAP_TAG_ was incubated for 5, 30, and 60 min with BM, and as shown in Figure 1C, the maximal binding of (H_6_)-GFP-SLAP_TAG_ to BM was reached very rapidly (5 min), showing no significant increments in its association at longer time points. These results indicated that the SLAP_TAG_ binds very rapidly and with an apparent high affinity to the BM. To quantify the affinity of the interaction between the SLAP_TAG_ with the BM, an adsorption isotherm was performed to determine the apparent equilibrium dissociation constant as described in the Materials and Methods section. As shown in Figure 1D, the apparent K_D_ was estimated as 4.7 μM. Interestingly, this dissociation constant value was in the same order as other K_D_ described for different microbial S-layer proteins and their respective bacterial cell walls (Garduno et al., 1995; Mader et al., 2004; Li et al., 2009). In addition, the maximum adsorption capacity (Bmax) of BM was estimated as 1.152 mmol of (H6)-GFP-SLAPTAG or 53.9 mg of protein per milliliter of BM or 0.0815 g of SLAPTAG protein/g of wet BM.

### Elution of (H_6_)-GFP-SLAP_TAG_ protein can be performed with different buffers

**Figure 2:**
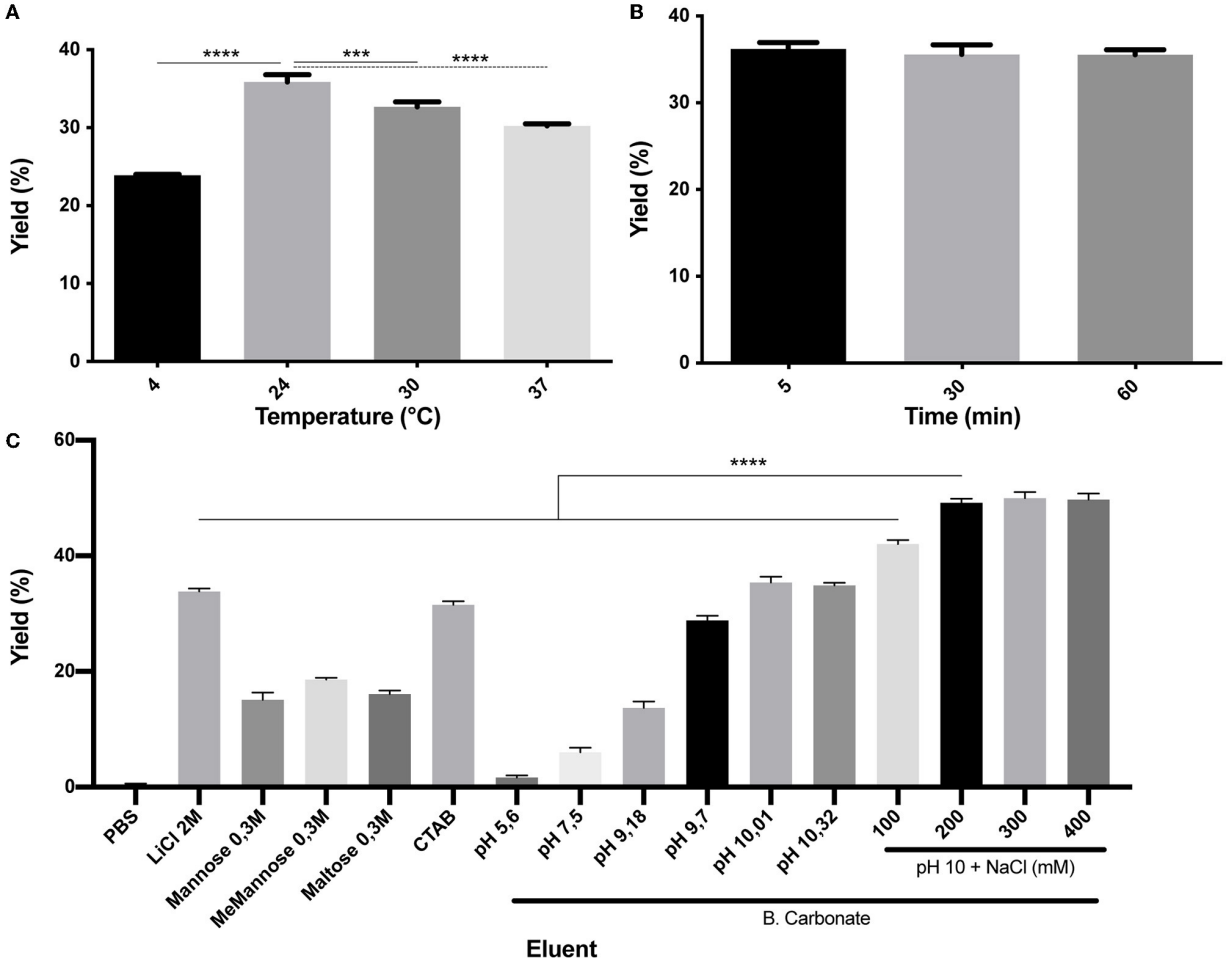
Characterization of (H_6_)-GFP-SLAP_TAG_ elution. **(A)** Figure compares (H_6_)-GFP-SLAP_TAG_ purification yield of SLAP_TAG_-based affinity chromatography for different temperatures of elution. **(B)** Figure compares (H_6_)-GFP-SLAP_TAG_ purification yield of SLAP_TAG_-based affinity chromatography for different times of elution incubation. **(C)** Figure compares (H_6_)-GFP-SLAP_TAG_ purification yield of SLAP_TAG_-based affinity chromatography system for different eluents. The asterisk denotes significant differences using the ANOVA method, the Bonferroni test (***p = 0,0003; ****p < 0.0001).

As it was mentioned, the SLAP_TAG_ contains the protein region responsible for the association of SlpA to the *L. acidophilus* cell wall. As reported, *L. acidophilus* SlpA has the natural ability to self-assemble on the bacterial surface to generate a proteinaceous layer named S-layer (Lortal et al., 1992), a highly ordered wall structure that functions as a protective barrier against bacteriophages, resistance to low pH and proteases, and bacterial adhesion. It was characterized that removal of the S-layer can be performed efficiently by the addition of chaotropic agents like LiCl, a compound that disrupts hydrogen bonds leading to a partial denaturation of proteins and the consequent detachment of the S-layer (Lortal et al., 1992). To study (H_6_)-GFP-SLAP_TAG_ elution from BM, LiCl solution was selected as the positive control. As shown in Figure 2A while PBS has no effect on protein elution, the detachment of BM-associated (H_6_)-GFP-SLAP_TAG_ was achieved with high efficiency with a 2M LiCl solution at room temperature, in a short lapse of 5 min (Figure 2B). As it was mentioned above, lithium solutions have certain deleterious effects on proteins, which is an undesired effect for protein purification, especially for enzyme purification. Therefore, to elute SLAP_TAG_-tagged proteins preserving protein structure and therefore their function, different milder alternatives of buffers were explored. As shown in Figure 2C, a series of sugars (monosaccharide and disaccharides) described previously (Fina Martin et al., 2019) were tested for the elution of the SLAP reporter observing only a partial elution efficiency when compared with the LiCl elution control (Figure 2C). Considering that the cationic nature of the SLAP_TAG_ at neutral pH is critical for the interaction of SLAP_TAG_ with its membrane-bound ligand, the teichoic acid, a set of cationic compounds were tested for the elution. As shown in Figure 2C, 0.3M of CTAB was able to elute (H_6_)-GFP-SLAP_TAG_ with similar efficiency as the LiCl solutions. Although CTAB was very efficient in this step, we found that this compound was difficult to remove downstream from the elution fraction. Then, taking into consideration that SLAP_TAG_ has a theoretical isoelectric point (pI) value of 9.92, consequently, we explored whether the modification of pH can be adapted as an elution method. As shown in Figure 2C, when the pH of the carbonate-bicarbonate buffer was close to the theoretical SLAP_TAG_ isoelectric point (pI), (H_6_)-GFP-SLAP_TAG_ was efficiently eluted from BM. In addition, after establishing the carbonate-bicarbonate buffer at pH 10 as an elution buffer, we explore whether the addition of NaCl can improve the elution process. As shown in Figure 2C, the addition of 200 mM of NaCl was able to maximize the yield of recovery of (H_6_)-GFP-SLAP_TAG_ in the eluate. Interestingly, at pH 7.6, the same concentration of NaCl (present in the binding/washing buffer) has no effect on detaching SLAP-tagged proteins from BM.

### The optimized SAC protocol allowed the efficient purification of proteins with similar efficiency to the high-performance Ni^2+^-charged agarose matrix (IMAC)

**Figure 3:**
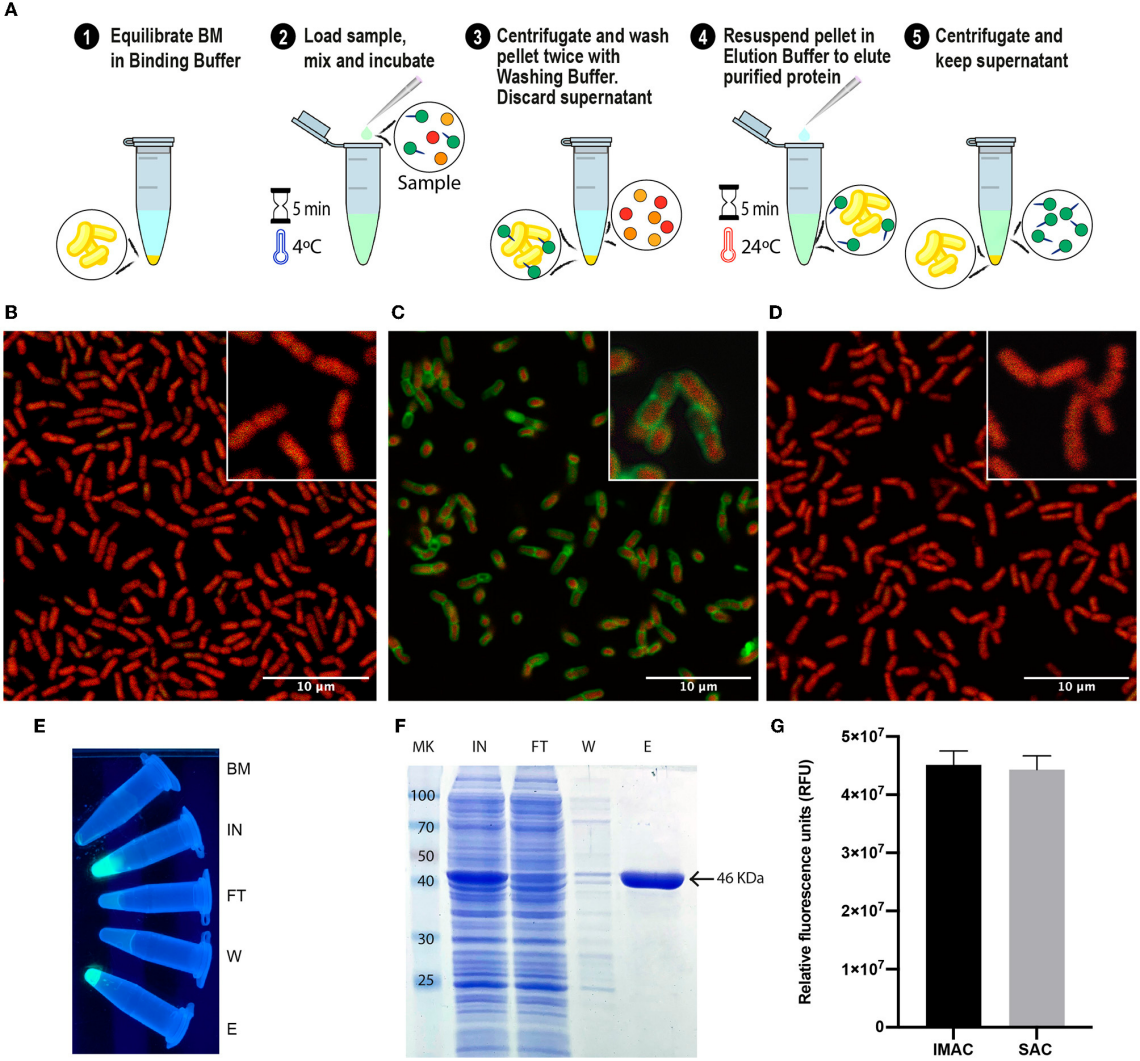
Optimized protocol for (H_6_)-GFP-SLAP_TAG_ purification using the Bio-Matrix and comparison with the high-performance Ni^2+^-charged agarose matrix. **(A)** Graphical description of the SLAP_TAG_-based affinity chromatography protocol. **(B)** Confocal microscopy of the Bio-Matrix. *Bacillus subtilis* red native autofluorescence is observed. **(C)** Confocal microscopy of (H_6_)-GFP-SLAP_TAG_ bound to the Bio-Matrix. GFP is visualized on the *Bacillus* surface. **(D)** Confocal microscopy of the Bio-Matrix after elution of (H_6_)-GFP-SLAP_TAG_. **(E)** Tubes containing purification fractions seen under UV light. GFP input fluorescence is recovered in the eluate. **(F)** Coomassie Blue staining analysis of (H_6_)-GFP-SLAP_TAG_ scaled purification. BM, Bio-Matrix; MK, protein marker (kDa); IN, input; FT, flow-through; W, wash; E, elution. **(G)** Immobilized metal affinity chromatography (IMAC) and SLAP_TAG_-based affinity chromatography systems are compared in their capacity to purify (H_6_)-GFP-SLAP_TAG_. Relative fluorescence of total (H_6_)-GFP-SLAP_TAG_ recovered in elution fraction is shown.

With all the experimental information obtained, an optimized SAC protocol for the purification of SLAP-tagged proteins was established as shown in Figure 3A. Remarkably, the entire process of protein purification can be performed in 15 min. To have a direct observation of the purification process of (H_6_)-GFP-SLAP_TAG_, the BM was fixed and immobilized on coverslips at different steps of the process of protein purification to be observed by confocal microscopy. As shown in Figure 3B, before the incubation of BM with (H_6_)-GFP-SLAP_TAG_, only a red native autofluorescence was observed from fixed *Bacillus subtilis* natto cells present in BM. Remarkably, after the incubation of BM with (H_6_)-GFP-SLAP_TAG_, it was possible to detect the adhesion of the reporter protein to the BM establishing a recombinant fluorescent S-layer that covers completely the bacterial surface (Figure 3C). As expected, after the addition of the elution buffer, the (H_6_)-GFP-SLAP_TAG_ was completely removed from the bacterial surface (Figure 3D). In addition, the whole process of purification can also be monitored by direct observation under UV light (Figure 3E).

Since SAC was efficient in protein purification, we compared this new technique against an established affinity purification protocol. For this, we took advantage of (H_6_)-GFP-SLAP_TAG_ which also has a His-tag that can be purified by a metal affinity chromatography or IMAC. As described in Materials and Methods, a bacterial lysate (H_6_)-GFP-SLAP_TAG_ was divided into two fractions, and both protocols were performed accordingly in parallel, confirming that SAC optimized protocol was able to purify (H_6_)-GFP-SLAP_TAG_ with high efficiency (Supplementary Table 1; Figure 3F) and with a similar yield to the one obtained with the commercial IMAC (Figure 3G).

### Proteins of different biological sources, molecular weights, biochemical functions, or expressed by prokaryotic or eukaryotic expression systems can be efficiently purified by the SAC

**Figure 4:**
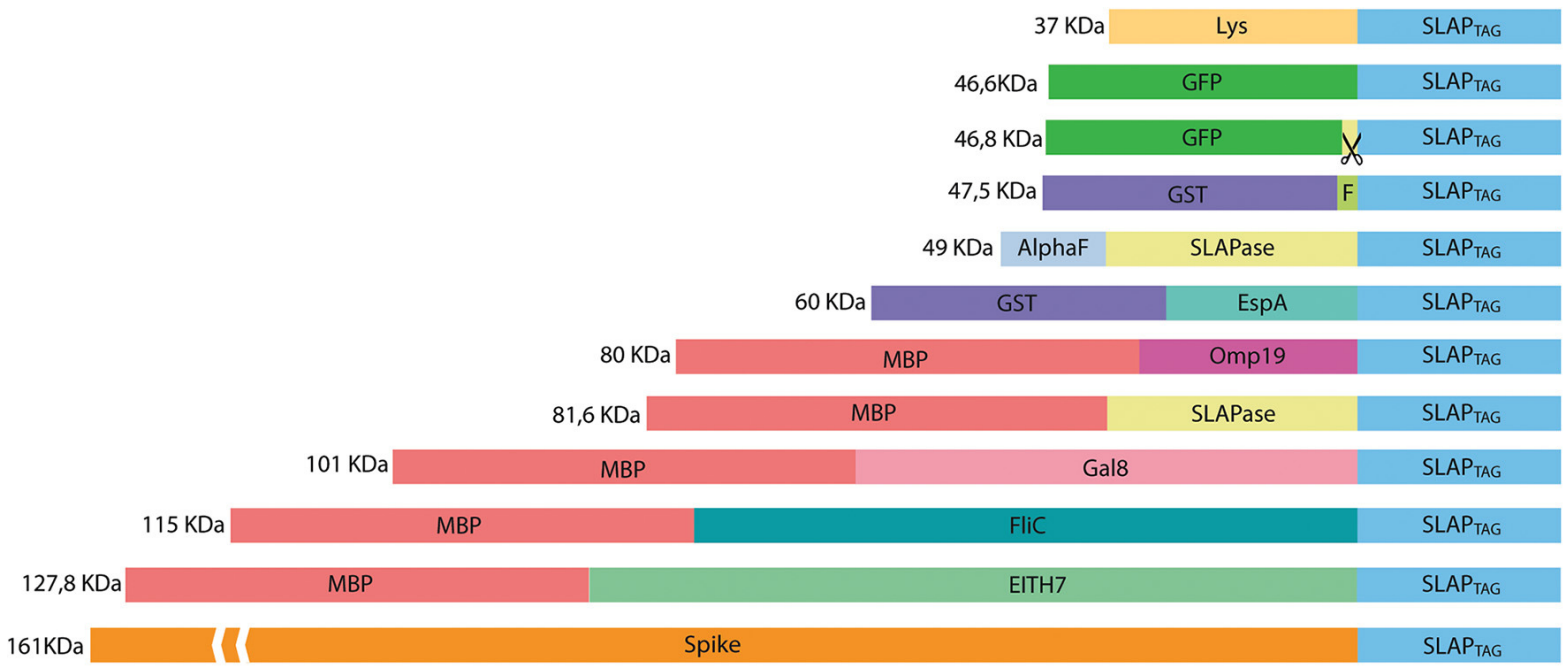
Representation of SLAP-tagged recombinant protein structure. Lys, bacteriophage T4 lysozyme; GFP, *Aequorea victoria* green fluorescent protein; GST, glutathione-s-transferase; HRV-3c, human Rhinovirus 3C Protease; EspA, *E. coli* EspA protein; Omp19, *B. abortus* Omp19 protein; MBP, maltose-binding protein; Gal8, mouse Galectin-8; EITH7, EspA, Intimin and Tir fusion protein from the Shiga toxin-producing *E. coli*; Spike, SARS-CoV-2 spike protein.

To evaluate whether SLAP_TAG_ and SAC can be adapted as a universal protein purification system, a set of the selected proteins were fused to the SLAP_TAG_ (Figure 4).

**Figure 5:**
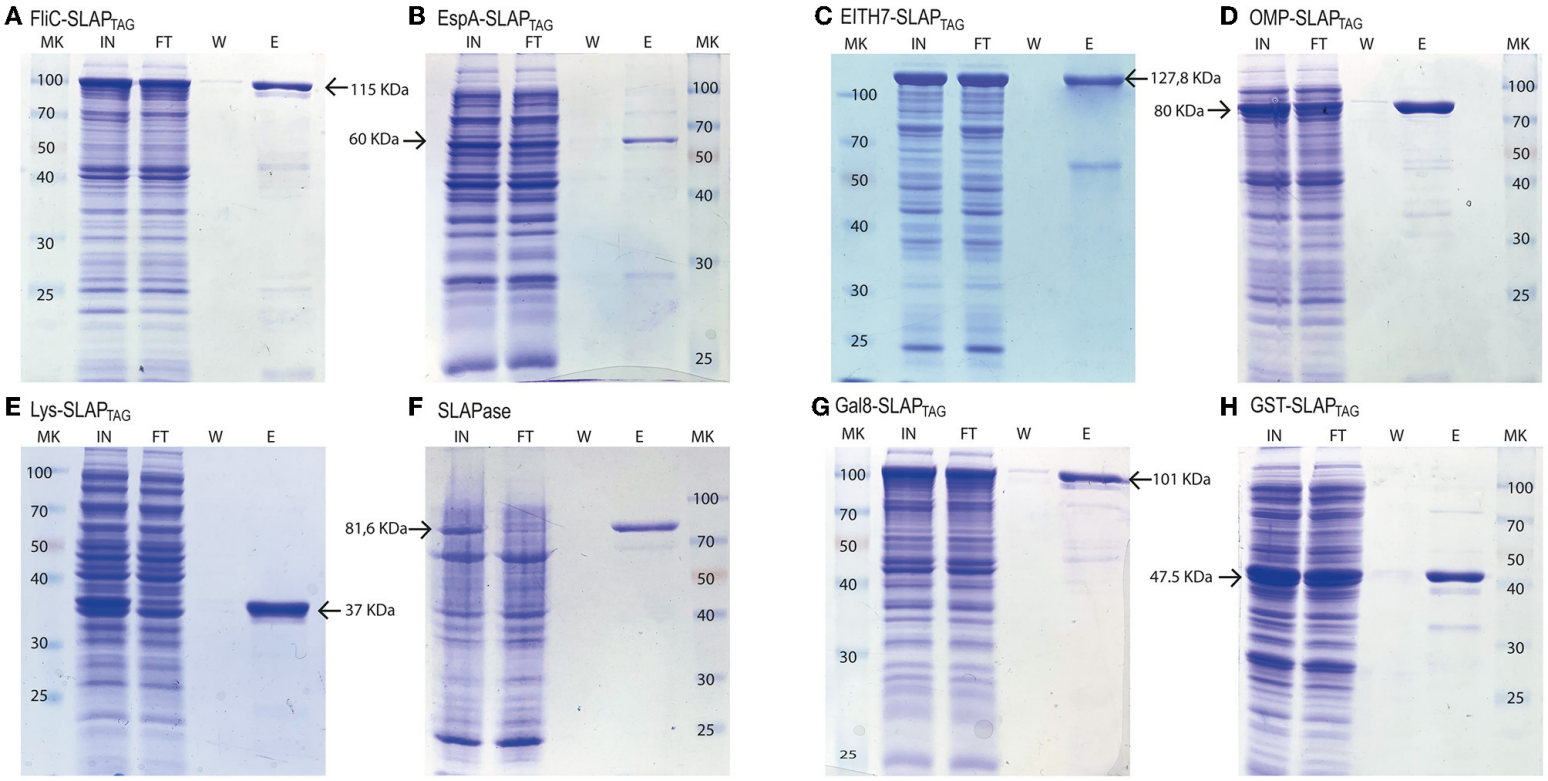
Analysis of different SLAP-tagged protein purifications expressed in *E. coli*. Coomassie Blue stained SDS-PAGE of purification fractions of SLAP_TAG_-based affinity chromatography for **(A)** FliC-SLAP_TAG_, **(B)** EspA-SLAP_TAG_, **(C)** EITH7-SLAP_TAG_, **(D)** Omp19-SLAP_TAG_, **(E)** Lys-SLAP_TAG_, **(F)** SLAP_ASE_ (human rhinovirus 3c fused to SLAP_TAG_), **(G)** Gal8-SLAP_TAG_, and **(H)** GST-SLAP_TAG_. MK, protein marker (kDa); IN, input; FT, flow-through; W, wash; E, elution.

Thus, bacterial proteins such as *Salmonella* flagellin (Figure 5A), the STEC proteins, EspA (Figure 5B), the chimeric EIT (Figure 5C), the *B. abortus* Omp19 (Figure 5D), the bacteriophage protein T7 lysozyme (Figure 5E), the human viral proteins Rhinovirus 3C Protease (Figure 5E), the mouse Galectin-8 (Figure 5G), and the commercial molecular tag GST (Figure 5H) were fused in-frame with the SLAP_TAG_.

**Figure 6:**
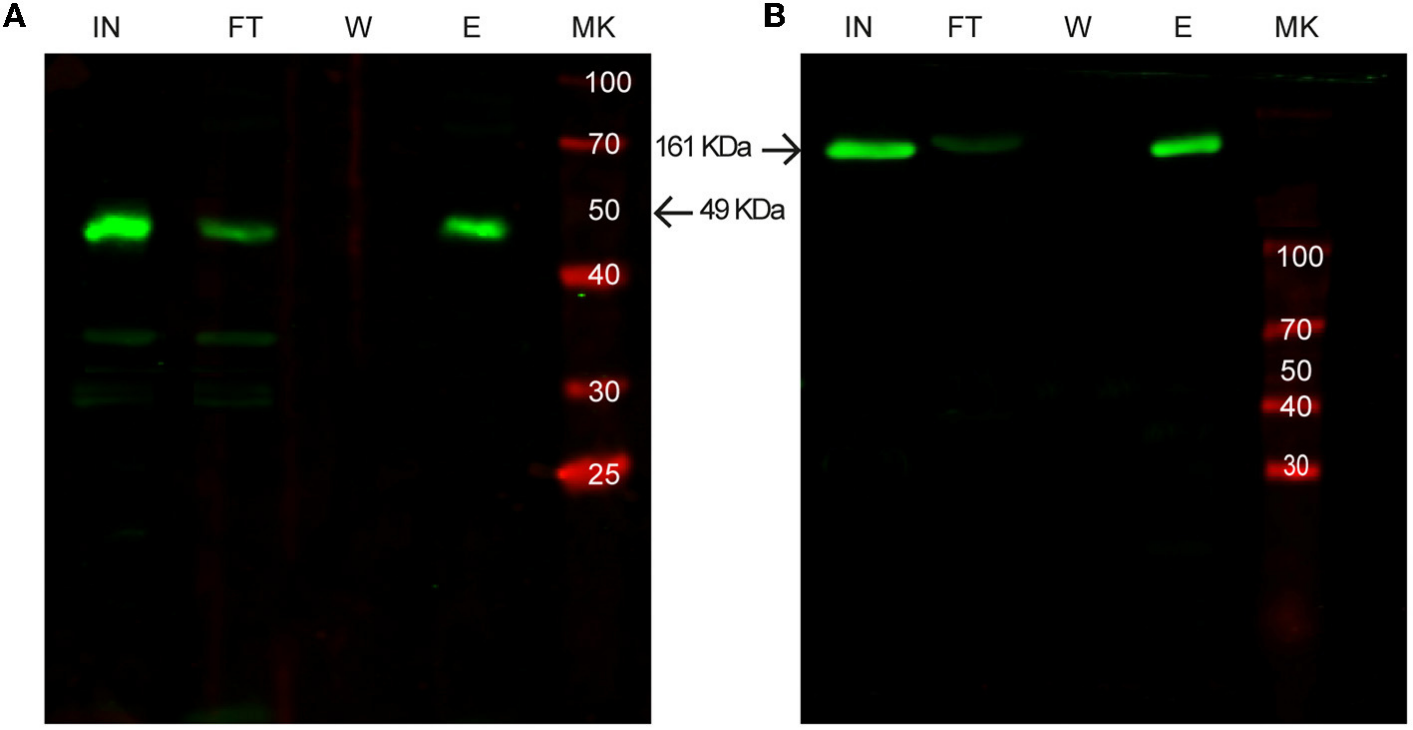
Western blot analysis of purification of SLAP-tagged proteins using different expression systems. **(A)** Purification of SLAP_ASE_ (human rhinovirus 3c fused to SLAP_TAG_) protease expressed in *Pichia pastoris*. **(B)** Purification of SARS-CoV-2 Spike-SLAP_TAG_ protein expressed in HEK293 cells. MK, protein marker (kDa); IN, input; FT, flow-through; W, wash; E, elution.

As shown in Figure 5, all the selected proteins were purified efficiently with SAC. In addition to the bacterial expression system, different protein expressions systems (yeast and mammalian cells) were also evaluated (Figure 6). As shown in Figure 6A, the viral HRV-3C protease fused to the SLAP_TAG_ was expressed and purified from *Pichia pastoris* supernatant. In addition, the SARS-CoV-2 SPIKE fused to the SLAP_TAG_ was purified from the supernatant of transfected HEK293 (Figure 6B). These results confirmed that the SLAP_TAG_ can be widely adopted for affinity chromatography purification. It is interesting to note that the protein SPIKE-SLAP_TAG_ was efficiently purified from a sample with a high protein content, such as a culture medium containing FBS, using SAC (Supplementary Figure 2). These findings indicate that SAC can be potentially employed as a first step to enrich a protein of interest directly from an unclarified feedstock derived from a host cell culture medium in downstream protein production processes.

### BM is a reusable chromatography matrix with long-term stability

**Figure 7:**
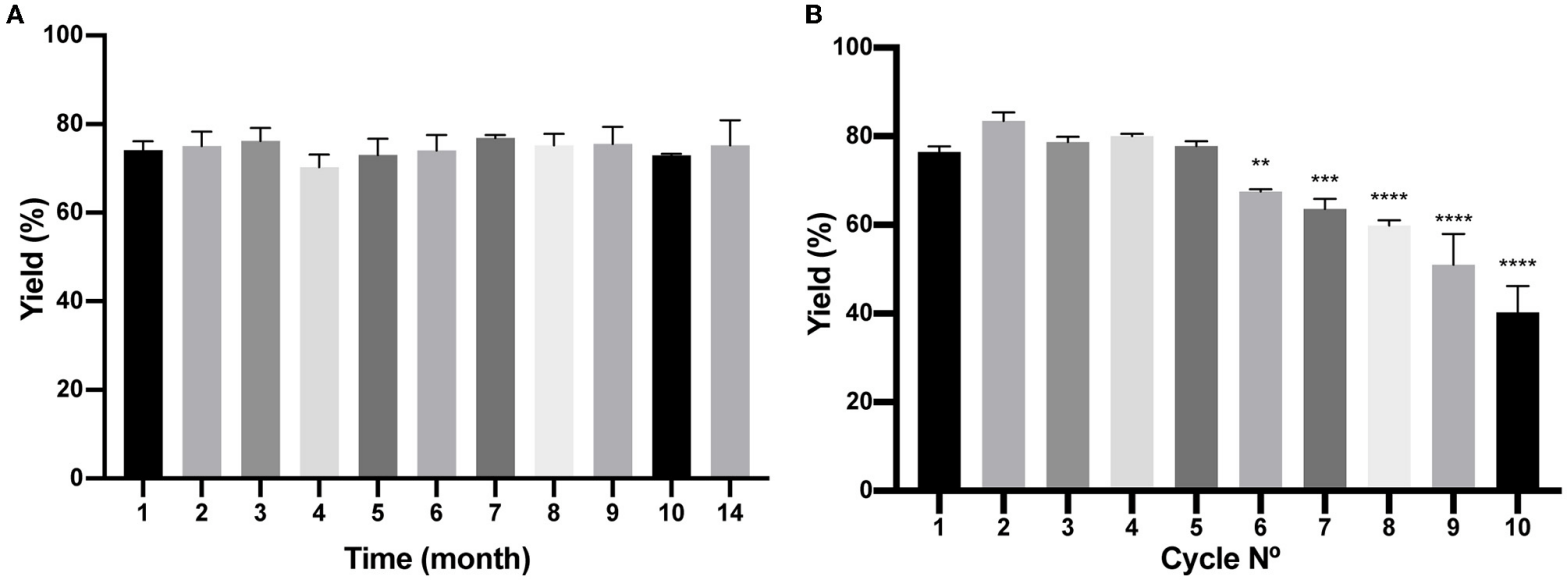
Analysis of Bio-Matrix stability in time and reuse. **(A)** The figure compares (H_6_)-GFP-SLAP_TAG_ purification yield of SLAP_TAG_-based affinity chromatography at different times when it is conserved at−20°C. **(B)** Figure compares (H_6_)-GFP-SLAP_TAG_ purification yield of SLAP_TAG_-based affinity chromatography for different cycles of reuse. The asterisk denotes a significant difference using the ANOVA method, Bonferroni test (**p = 0,0059; ***p = 0,0002; ****p < 0.0001)

As described in the Materials and Methods section, a batch of BM was produced, and several aliquots were frozen at −20°C to study time stability. As shown in Figure 7A, at different times, a few aliquots were unfrozen and tested for protein purification of the reporter protein (H_6_)-GFP-SLAP_TAG_ being the BM stable for more than a year that was tested (14 months). In addition, BM reusability capacity was evaluated determining that the matrix can be reused five times with no modification of the protein purification yield (Figure 7B).

### Use of the Human Rhinovirus 3C protease fused to the SLAP_*TAG*_ (SLAP_ASE_) to release the SLAP-tagged proteins from BM

**Figure 8:**
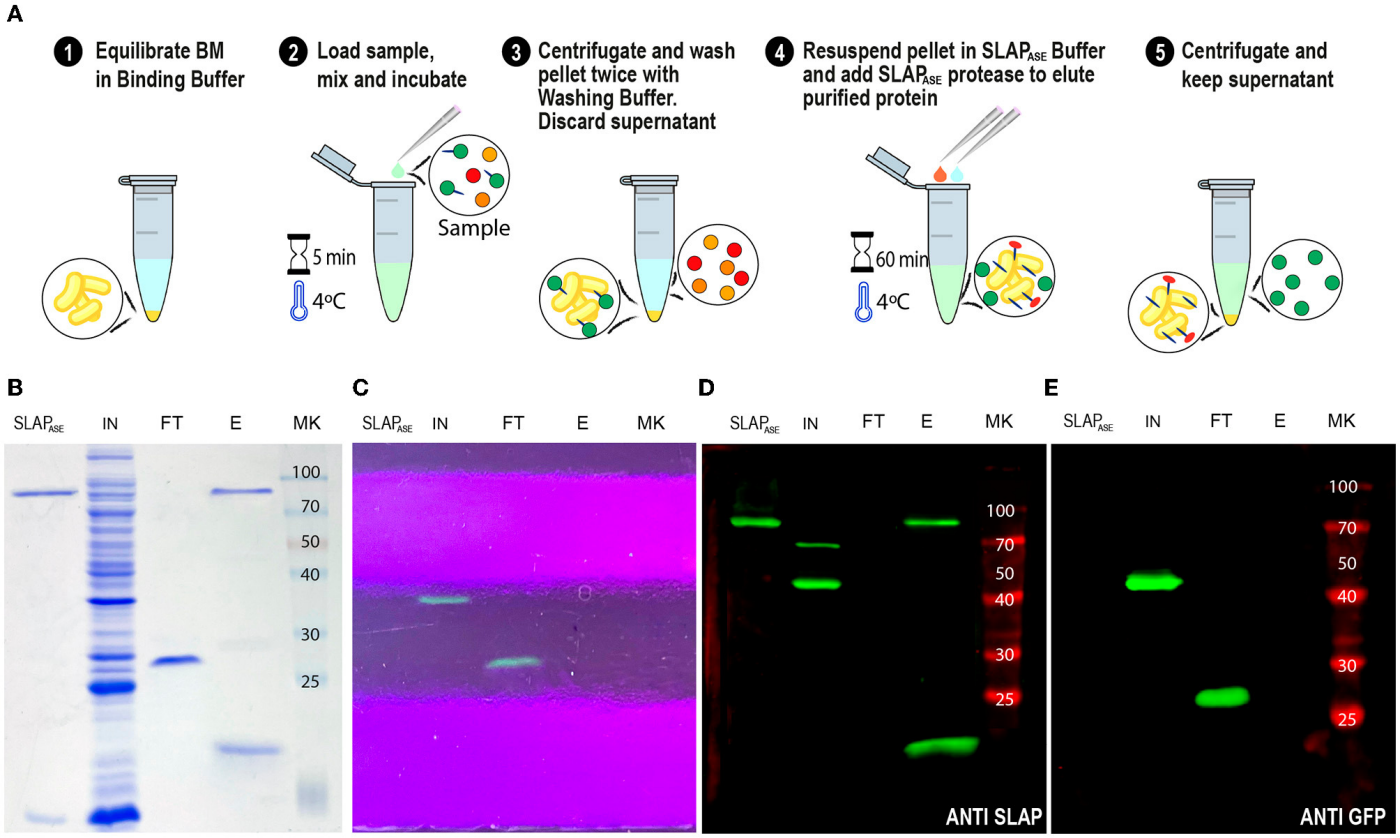
SLAP_TAG_ removal. **(A)** Graphical description of the protocol to remove the SLAP_TAG_ using SLAP_TAG_-based affinity chromatography. SDS-PAGE of purification fractions of SLAP_TAG_ removal protocol stained with Coomassie Blue solution **(B)** or under UV light **(C)**. Western Blot of purification fractions of SLAP_TAG_ removal protocol revealed with anti-SLAPTAG **(D)** or anti-GFP **(E)** antibodies. SLAP_ASE_, purified protease; IN, input; FT, flow-through; E, elution; MK, protein marker (KDa).

As shown in Figures 4, 5, the gene sequence of the viral protease HRV-3C was fused to the SLAP_TAG_ (named SLAP_ASE_), recombinantly expressed, and purified (Figures 8B, D, lane SLAPase) to evaluate its activity. A reporter protein for SLAP_ASE_ activity was generated (GFP-LEVLFQGP-SLAP_TAG_) (Table 1), and a protocol for tag removal by SLAPase was set as described in the Methods section. As shown in Figure 8A, the GFP-LEVLFQGP-SLAP_TAG_ was expressed (Figures 8B, C, lane Input or IN) and mixed with the BM for 5 min allowing the binding process. After binding, a purified SLAP_ASE_ was added to the mix and incubated for 60 min at 4°C, releasing a tag-less GFP (Figures 8B, C, E, lanes flow-through or FT) by proteolysis. Cut SLAP_TAG_ is not observed in the flow-through indicating that was retained by the BM along with the SLAPase. To confirm these steps, post-proteolysis BM was incubated with LiCl solution to recover any residual bound SLAP-tagged protein. As shown in Figures 8B, D (lane Elution or E), the LiCl solution released the SLAPase and the cut SLAP_TAG_.

### Adaptation of SAC to magnetic affinity chromatography

**Figure 9:**
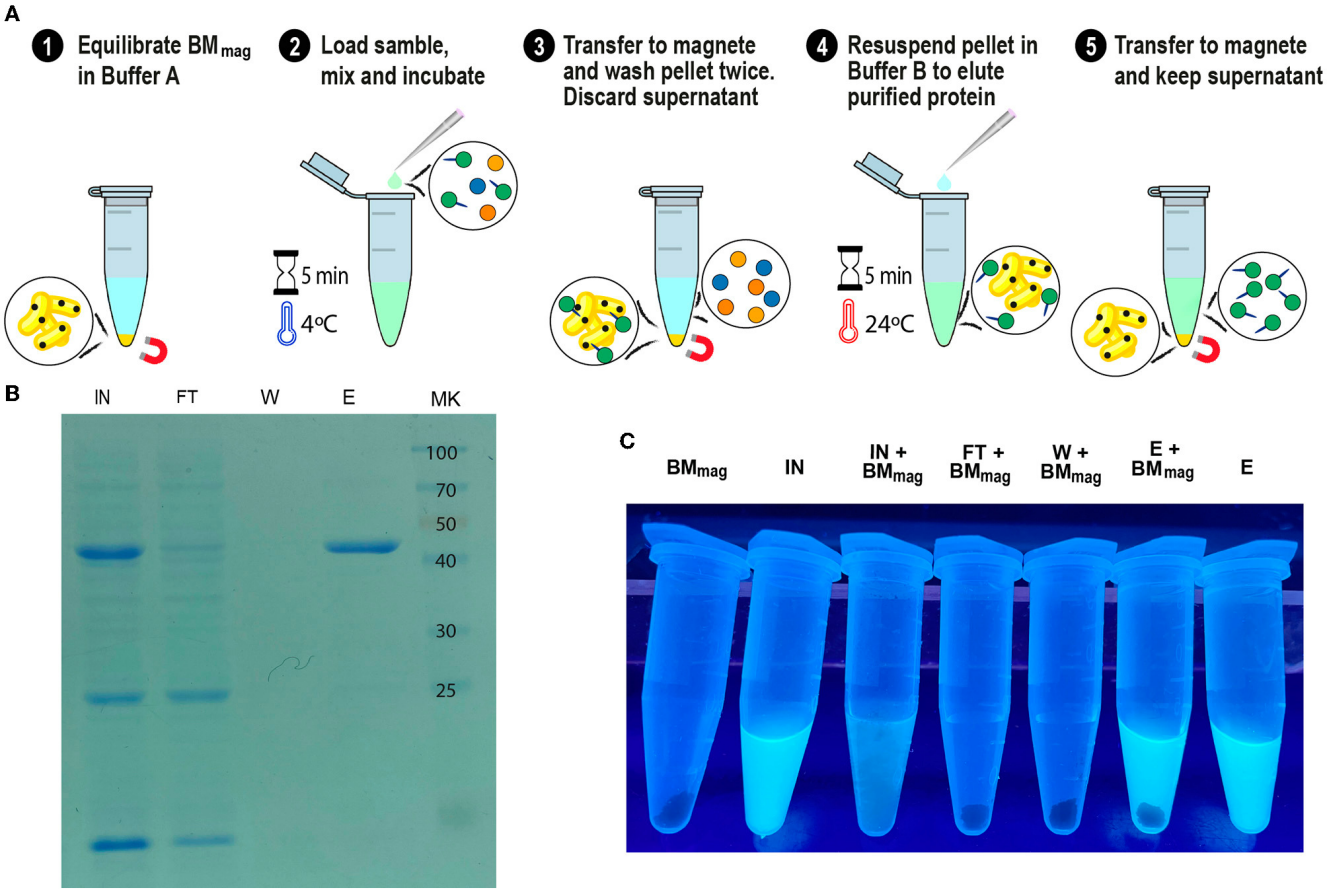
GFP-SLAP_TAG_ purification using Magnetic Bio-Matrix. **(A)** Graphical description of the purification protocol for magnetic Bio-Matrix. **(B)** Photograph of 1.5 ml tubes containing fractions of GFP-SLAP_TAG_ magnetic purification under UV light. BM_mag_, magnetic Bio-Matrix; IN, input; FT, flow-through; W, wash; E, elution. **(C)** Tubes containing magnetic purification fractions are seen under UV light. GFP input fluorescence is recovered in the eluate.

Since we confirmed the efficiency and universality of SAC for the purification of recombinant proteins, we explored a magnetic affinity chromatography alternative for SAC (Figure 9A). As shown in Figures 9B, C, and Supplementary Video 1, the (H_6_)-GFP-SLAP_TAG_ was purified in a few and easy steps. As shown in the SDS-PAGE (Figure 9B), the adapted SAC protocol for magnetic chromatography (BM_mag_) was able to purify the reporter protein (H_6_)-GFP-SLAP_TAG_ very efficiently (Figure 9B, lane Elution or E). As shown in Supplementary Table 2, BMmag is identical to BM in terms of purification performance. These results indicate that the binding capacity of BM is not modified by its association with magnetic particles.

Interestingly, this protocol can be adapted to commercial devices that use magnetic force for protein or DNA purification (like King Fisher Flex from Thermo Fisher). In Figure 9C, the entire process of purification was monitored by direct observation under UV light (Supplementary Video 1). Noteworthy, the binding of the BM_mag_ to GFP quenched the fluorescence of this protein, an effect that was described for transition metal binding to GFP or binding of iron cations to fluorescent proteins (Figure 9C, tube IN+BM_mag_) (Richmond et al., 2000; Kim et al., 2021).

## Discussion

The *B. subtilis-derived* matrix, named here as Bio-Matrix (BM), showed a high performance in its binding and elution capacities, combined with good purification parameters for our reporter protein (H_6_)-GFP-SLAP_TAG_. The results presented here show that the SAC protocol can be potentially adapted as a universal tool for recombinant protein purification. Thus, proteins from different origins, with different molecular weights, or produced by different recombinant expression systems (bacteria, yeast, or cells) can be efficiently purified by SAC. In addition to its universal application, we demonstrate here that SAC was able to achieve a protein yield similar to those obtained by commercial affinity chromatography systems such as the immobilized metal affinity chromatography or IMAC. As reported, in protein production bioprocess, chromatography is the most expensive step (Wong et al., 2018). Of interest, the SAC protocol only uses simple and low-cost reagents, consequently presenting an economic advantage in protein purification over current commercial systems. In addition, since the Bio-Matrix is “grown” instead of being chemically synthesized, the production of larger quantities of chromatography matrix can be achieved easily by simply scaling up the volume of the bioreactor. In addition, small research laboratories can easily produce an in-house BM version by the protocol provided here.

One critical step in the generation of the affinity matrix is the immobilization of the affinity ligand to the chromatography matrix. Initially, at the beginning of the affinity chromatography development, the immobilization of affinity ligands was performed by covalent modification using diazo coupling (Rodriguez et al., 2020). This procedure allows to immobilize different haptens or certain proteins to isolate antibodies. A second breakthrough in affinity chromatography was the development of the cyanogen bromide (CNBr) immobilization method which allows the easy cross-link of proteins or peptides to the activated agarose matrix (Rodriguez et al., 2020). These two major advances were combined in 1969 by Cuatreacasas et al. where the term affinity chromatography was used for the first time (Cuatrecasas et al., 1968).

In contrast, in our approach, the affinity ligands (LTA and teichoic acid) are naturally integrated into the surface of the BM (Lortal et al., 1992), and consequently, no chemical reactions to cross-link the affinity ligands are required. Remarkably, no toxic chemicals or solvents are required for BM production.

The protein purification procedures described in this study were conducted in a batch format, utilizing either centrifugation or magnetic separation for the various steps of the process. Although SAC in a batch format proved to be a very efficient and straightforward method, the adaptation of SAC to the column format was more cumbersome because BM tended to clog the column, resulting in slow flow rates. To address this limitation, we explored the immobilization of BM on various supports that allow high-flow chromatography, such as polyurethane sponges, polystyrene beds, glass beads, and cellulose, but with limited success (data not shown). Additionally, we investigated different potential chromatography materials that can directly bind SLAP_TAG_-tagged proteins, which could potentially be useful for SAC in the column format. Our preliminary findings indicate that chitosan is a promising candidate (Supplementary Figure 3).

One outstanding aspect of SAC was its ability to efficiently capture SLAP-tagged proteins from a high-concentration protein solution, such as an unclarified crude sample (e.g., HEK295 cell supernatant) (Supplementary Figure 2). Importantly, this characteristic allows us to propose adapting SAC for expanded-bed adsorption (EBA) chromatography (Chase, 1994; Hjorth et al., 1998). EBA has been demonstrated to be a useful method, particularly for protein capture in a continuous protein purification process from unclarified feedstocks in the recovery of enzymes and therapeutic proteins from a variety of expression hosts, without the need for extensive clarification steps (Schneider et al., 2022). Interestingly, a magnetic version of EBA (EBA/MSFBs) has been explored (Tong and Sun, 2003). Magnetically susceptible chromatography supports are forced to low back-mixing by applying a weak, external magnetic field that oriented the magnetic particles axially or transversely relative to the flow (Schneider et al., 2022).

As shown here, BM was able to efficiently capture SLAP-tagged proteins directly from different feeds such as bacterial lysates, *Pichia pastoris* supernatant, or HEK293 cell supernatant in a single step (Figures 3, 5, 6; Supplementary Figure 2). In addition, a magnetically susceptible Bio-Matrix (BMmag) was generated that showed the same purification properties than BM (Supplementary Tables 1, 2). Both results made SAC an interesting technique for a potential EBA or EBA/MSFBs adaptation.

In the last decade, the introduction of single-use technologies has enlightened the potential for reduced regulatory and operational costs associated with chromatography. The researchers point to its potential for simpler operation, shorter processing times, and decreased buffer consumption, leading to better economics (Hester et al., 2021). In addition, the lack of need for cleaning over repeat-use cycles significantly reduces costs. SAC could be potentially adapted as a single-use alternative chromatography for some industries, with the benefit of being more eco-friendly than those available on the market, as it is a biologically based and biodegradable matrix.

SAC proved to successfully adapt to protease tag removal. Although new technologies are being developed for tag removal (e.g., inteins), the enzymatic cleavage of the tag is still preferred as it is the most controlled process, with no premature cleaving and the best yields are obtained (Pina et al., 2014). Moreover, new technologies might be compatible with SAC.

Therefore, we propose SLAP_TAG_ affinity chromatography for protein purification in industries with permissive regulations. SAC can be adapted as an in-house protein purification system, available for any laboratory around the globe, to produce pure recombinant proteins for research, diagnosis, and the food industry. Although so far regulatory issues might preclude the use of the SAC for proteins used as biotherapeutics, new efforts have been performed to develop a new version of matrix chromatography suitable for more stringent industrial or clinical purposes.

## Data availability statement

The original contributions presented in the study are included in the article/Supplementary material, further inquiries can be directed to the corresponding authors.

## Ethics statement

The experimental procedure of this study (Permit Number CICUAE UNSAM 15/2018) was approved by the Committee on the Ethics of Animal Experiments of the Universidad Nacional de San Martín (UNSAM), under the recommendations for animal experimentation (Helsinki Declaration and its amendments, Amsterdam Protocol of Welfare and Animal Protection and National Institutes of Health, USA NIH, Guidelines: Guide for the Care and Use of Laboratory Animals).

## Author contributions

EM performed most of experimental work and also generate figures and contributed to discussion of manuscript. PU performed molecular cloning of multiples proteins used. GE perform experiments regarding mammalian cells proteins expression. MP perform microscopy results. CD perform *Pichia pastoris* experiments. MR contribute in the manuscript writing and results discussion of the manuscript. GB is the P.I. and director of the project, contributing to experiment design, result discussion, and writing of the manuscript. All authors contributed to the article and approved the submitted version.
