# Supplementary material for: Adaptation of the binding domain of Lactobacillus acidophilus S-layer protein as a molecular tag for affinity chromatography development
Source: Front Microbiol. 2023 Jun 13;14:1210898. doi: 10.3389/fmicb.2023.1210898 (PMC10293925; doi:10.3389/fmicb.2023.1210898)
Supplement: Supplementary file 1 [file Image_1.PDF]

Lactobacillus acidophilus SlpA protein.

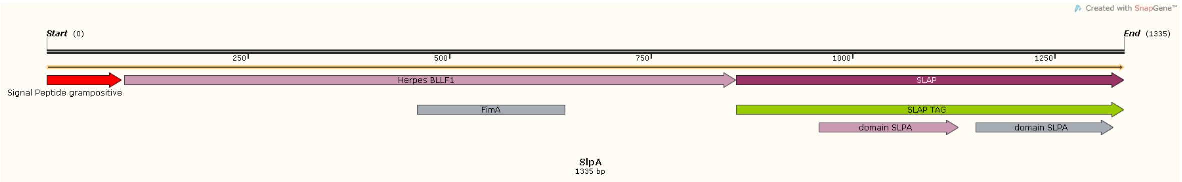


```
1   mkknlrivsa aaallavapv aasavstvsa attinasssa intntnakyd vdvtpsvsav
61  aavaantann tpaiagnltg tisasyngkt ytanlkadte natitaagst tavkpaelaa
121 gvaytvtvnd vsfnfgsena gktvtlgsan snvkftgtns dnqtetnvst lkvkldqngv
181 asltnvsian vyainttdns nvnfydvtsg atvtngavsv nadnqgqvnv anvvaainsk
241 yfaaqyadkk lntltanted aikaalkdqk idvnsvgyfk aphtftvnvk atsntngksa
301 tlpvvvtvpn vaeptvasvs krimhnayyy dkdakrvgtd svkrynsvsv lpntttingk
361 tyyqvvengk avdkyinaan idgtkrtlkh nayvyasskk rankvvlkkg evvttygasy
421 tfkngqkyyk igdntdktyv kvanfr
```

Fig. Supp. 1: Amino acid sequence of SlpA *of Lactobacillus acidophilus*. The region called SLAP$_{TAG}$ is indicated in red, with the SLP-A domains underlined, as predicted by InterProScan.

SLAP$_{TAG}$:

```
        10         20         30         40         50         60
NVKATSNTNG KSATLPVVVT VPNVAEPTVA SVSKRIMHNA YYYDKDAKRV GTDSVKRYNS

        70         80         90        100        110        120
VSVLPNTTTI NGKTYYQVVE NGKAVDKYIN AANIDGTKRT LKHNAYVYAS SKKRANKVVL

       130        140        150
KKGEVVTTYG ASYTFKNGQK YYKIGDNTDK TYVKVANFR
```

Theoretical pI/Mw: 9.92 / 17591.93
