# Supplementary material for: Adaptation of the binding domain of Lactobacillus acidophilus S-layer protein as a molecular tag for affinity chromatography development
Source: Front Microbiol. 2023 Jun 13;14:1210898. doi: 10.3389/fmicb.2023.1210898 (PMC10293925; doi:10.3389/fmicb.2023.1210898)
Supplement: Supplementary file 2 [file Image_2.PDF]

Figure Supplementary 2

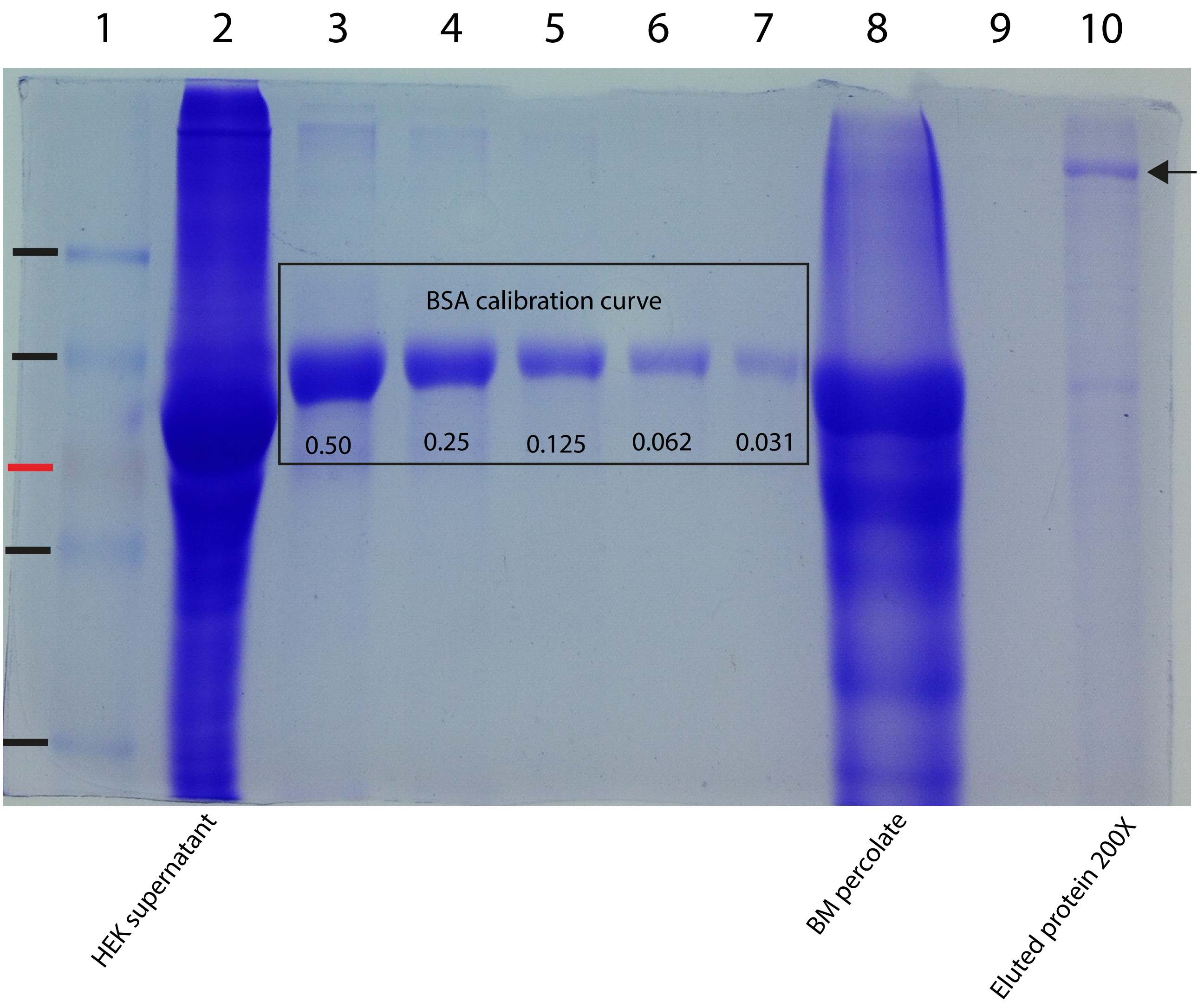

Fig. Supp. 2: The figure shows the process of purifying the protein SPIKE-SLAP from an unclarified feedstock derived from the supernatant of HEK293 cells transfected with a plasmid encoding the chimeric protein SPIKE-SLAP (lane 2). The arrow indicates the purified protein SPIKE-SLAP by BM (lane 10). The rectangle shows a BSA calibration curve used to estimate the amount of protein by comparing the intensity of the protein band (lanes 3-7). Lane 1 shows the STD molecular weight, with bands at 100 kDa, 70 kDa, 50 kDa, 40 kDa, and 30 kDa from top to bottom.
