# Supplementary material for: Adaptation of the binding domain of Lactobacillus acidophilus S-layer protein as a molecular tag for affinity chromatography development
Source: Front Microbiol. 2023 Jun 13;14:1210898. doi: 10.3389/fmicb.2023.1210898 (PMC10293925; doi:10.3389/fmicb.2023.1210898)
Supplement: Supplementary file 3 [file Image_3.PDF]

Figure Supplementary 3

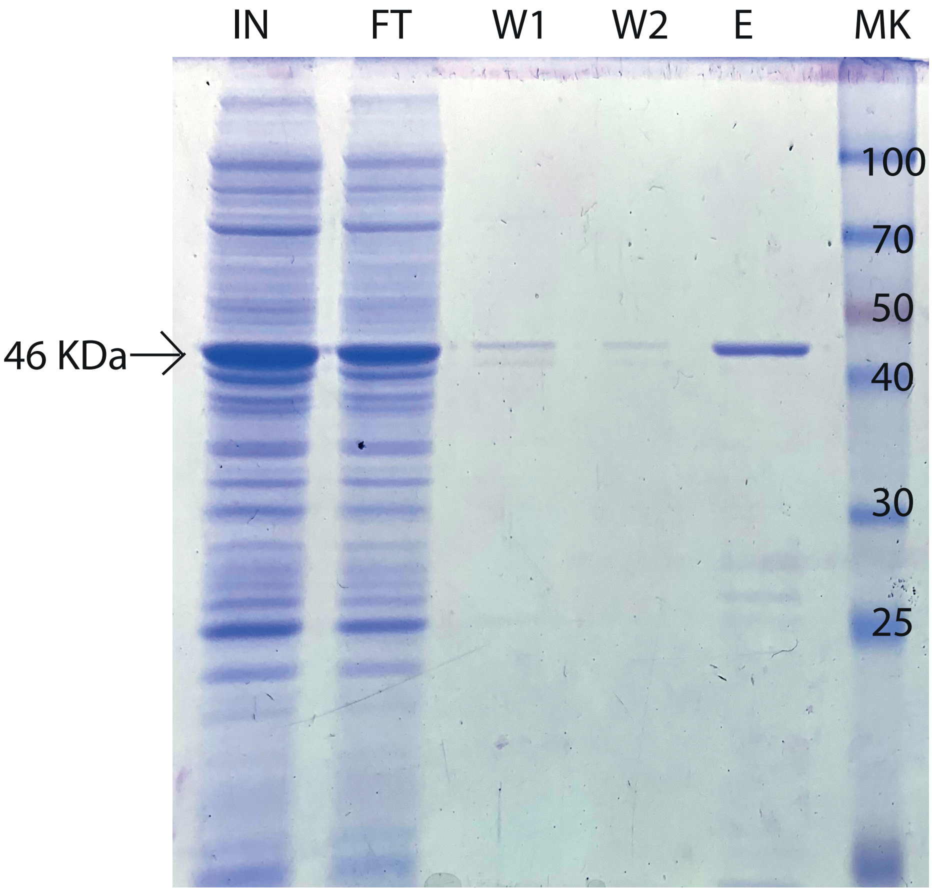

Figure Supp. 3: Coomassie Blue stained SDS-PAGE of purification fractions of (H6)-GFP-SLAPTAG using chitosan minispheres. IN = input; FT = flowthrough; W1 = first wash; W2 = second wash; E = elution; MK = protein marker (KDa).
