# Supplementary material for: Adaptation of the binding domain of Lactobacillus acidophilus S-layer protein as a molecular tag for affinity chromatography development
Source: Front Microbiol. 2023 Jun 13;14:1210898. doi: 10.3389/fmicb.2023.1210898 (PMC10293925; doi:10.3389/fmicb.2023.1210898)
Supplement: Supplementary file 4 [file Table_1.PDF]

Table Supp. 1: Purification table of GFP-SLAP$_{tag}$ using the Bio-Matrix

Step	Vol (ml)	Total protein (mg)	Total activity (AUF)	Specific Activity (AUF/mg)	Purification fold	Yield (%)	Efficiency
Crude extract	10	591	$3{,}75 \times 10^{11}$	$6{,}34 \times 10^{8}$	1	100	-
Biomatrix	1	5,96 ± 0,30	$2{,}96 \times 10^{11} \pm 1{,}57 \times 10^{10}$	$4{,}96 \times 10^{10} \pm 2{,}92 \times 10^{9}$	78,23 ± 4,61	78,90 ± 4,20	61,81 ± 6,33

Activity RFU relative fluorescence units
