# Supplementary material for: Adaptation of the binding domain of Lactobacillus acidophilus S-layer protein as a molecular tag for affinity chromatography development
Source: Front Microbiol. 2023 Jun 13;14:1210898. doi: 10.3389/fmicb.2023.1210898 (PMC10293925; doi:10.3389/fmicb.2023.1210898)
Supplement: Supplementary file 5 [file Table_2.PDF]

Table Supp. 2: Purification table of GFP-SLAP$_{tag}$ using the magnetic Bio-Matrix (BM$_{mag}$)

Step	Vol (ml)	Total protein (mg)	Total activity (AUF)	Specific Activity (AUF/mg)	Purification fold	Yield (%)	Efficiency
Crude extract	10	544,7	3,46 x10^{11}	6,34 x10^{8}	1	100	-
BM$_{mag}$	1	5,41 ± 0,52	2,66x10^{11} ± 2,56 x10^{10}	4,91 x10^{10} ± 4,15 x10^{9}	77,23 ± 6,61	76,95 ± 7,39	59,00 ± 5,72

Activity RFU relative fluorescence units
